# Reviewer acknowledgement 2013

**DOI:** 10.1186/1465-9921-15-7

**Published:** 2014-02-03

**Authors:** Jan Lötvall, Reynold A Panettieri

**Affiliations:** 1 University of Gothenburg, Krefting Research Centre, BOX 424, Göteborg, Sweden; 2 University of Pennsylvania, 423 Guardian Dr #175, Philadelphia, PA 19104, USA

## Abstract

**Contributing reviewers:**

The editors of *Respiratory Research* would like to thank all of our reviewers who have contributed to the journal in Volume 14 (2013).

## 

Ian Adcock

United Kingdom

Mikael Adner

Sweden

Bernard Aguilaniu

France

Alvar Agusti

Spain

Vassilis Aidinis

Greece

Muhammad Sajid Hamid Akash

Pakistan

Neil Alexis

United States of America

Stefano Aliberti

Italy

James Allinson

United Kingdom

Steven An

United States of America

Alessandro Antonelli

Italy

Raffaele Antonelli-Incalzi

Italy

Sabina Antoniu

Romania

Kjetil Ask

Canada

Sergei Atamas

United States of America

Paul Auer

United States of America

Charles Auffray

France

Mark Avdalovic

United States of America

Malin Axelsson

Sweden

David Badesch

United States of America

Mona Bafadhel

United Kingdom

Gregory Bagby

United States of America

Els Bakker

Netherlands

Vivek Balasubramaniam

United States of America

Peter Baluk

United States of America

Joan Albert Barbera

Spain

Esther Barreiro

Spain

Konstantinos Bartziokas

Greece

Erik Bathoorn

Netherlands

Frederic Batteux

France

Hasan Bayram

Turkey

Celine Beamer

United States of America

Richard Beasley

New Zealand

Juergen Behr

Germany

Christoph Beisswenger

Germany

John Kelley Bentley

United States of America

Jean-Francois Bernaudin

France

Ivan Bertoncello

Australia

Valerie Besnard

France

Vineet Bhandari

United States of America

Andras Bikov

Hungary

Colin David Bingle

United Kingdom

James Birchler

United States of America

Elyse Bissonnette

Canada

Lucie Blais

Canada

Francesco Blasi

Italy

Yury Bochkov

United States of America

Marcel Bonay

France

Ynuk Bosse

Canada

Apostolos Bossios

Sweden

Fernando Botelho

United States of America

Graham Bothamley

United Kingdom

Arnaud Bourdin

France

Jane Bourke

Australia

Demosthenes Bouros

Greece

Ken Bracke

Belgium

Fulvio Braido

Italy

Corry-Anke Brandsma

Netherlands

Danny Brazzale

Australia

Steven Brody

United States of America

Rossa Brugha

United Kingdom

Vito Brusasco

Italy

Dominique M A Bullens

Belgium

Pierre-Regis Burgel

France

Chris Burtin

Belgium

Derek Byers

United States of America

Peter Calverley

United Kingdom

Carlos Camargo

United States of America

Gianna Camiciottoli

Italy

Luigi Camporota

United Kingdom

Kai-Håkon Carlsen

Norway

Johnny Carson

United States of America

Edoardo Casiglia

Italy

Suzanne Cassel

United States of America

Vincent Castranova

United States of America

Didier Cataldo

Belgium

Mario Cazzola

Italy

Chung-Ming Chen

Taiwan

Yin Chen

United States of America

Anne Chetty

United States of America

Marco Chilosi

Italy

Dong-Sic Choi

South Korea

Kian Fan Chung

United Kingdom

Allan Coates

Canada

Harold Collard

United States of America

Gregory Conner

United States of America

Enrico Conte

Italy

Marco Contoli

Italy

Philip Cooper

United Kingdom

Ferry Cornelissen

Netherlands

Borja G Cosio

Spain

Vincent Cottin

France

Francis Couturaud

France

Harvey Coxson

Canada

Bruno Crestani

France

Gerard Curley

Ireland

Sven-Erik Dahlén

Sweden

Kaid Darwiche

Germany

Wilfried de Backer

Belgium

Frans de Jongh

Netherlands

Al de Weerd

Netherlands

Bennett Deboisblanc

United States of America

Monique Dehoux

France

Lien Dejager

Belgium

Stefano Del Giacco

Italy

Deepak Deshpande

United States of America

Fabiano Di Marco

Italy

Antonino Di Stefano

Italy

Quoc Thai Dinh

Germany

Ratko Djukanovic

United Kingdom

Claudia Dobler

Australia

James Dodd

United Kingdom

Claire Doerschuk

United States of America

Joanna Domagala-Kulawik

Poland

Claudio F Donner

Italy

James Donohue

United States of America

David Douda

Canada

Mark Dransfield

United States of America

Paul Dudas

United States of America

Steven Duncan

United States of America

Benedicte Durand

France

Balthasar Eberle

Switzerland

Ronald Eccles

United Kingdom

Carsten Ehrhardt

Ireland

Stuart Elborn

United Kingdom

Souheil El-Chemaly

United States of America

Ralph Epaud

France

David Erle

United States of America

Christopher Evans

United States of America

Leonardo Fabbri

Italy

Aurelie Fabre

Ireland

Lucy Fairclough

United Kingdom

David Fairlie

Australia

Laszlo Farkas

United States of America

Carol Feghali-Bostwick

United States of America

Renata Ferrari

Brazil

Gilbert Ferretti

France

Daniel Clark Files

United States of America

Bernie Fischer

United States of America

Paul Forsythe

Canada

Allison Fryer

United States of America

Trentham Furness

Australia

Marcelo Gama de Abreu

Germany

Jennie Gane

United Kingdom

Ravendra Garg

Canada

Kathy Gately

Ireland

Amiq Gazdhar

Switzerland

Moyar Qing Ge

United States of America

Arthur Gelb

United States of America

Steve Georas

United States of America

Maher Ghamloush

United States of America

Troy Ghashghaei

United States of America

Mark Giembycz

Canada

Stephan Glasser

United States of America

Elena Goncharova

United States of America

Reinoud Gosens

Netherlands

Harry Gosker

Netherlands

Philippe Gosset

France

Clive Grattan

United Kingdom

Florian Greten

Germany

Matthias Griese

Germany

Andreas Groeschel

Germany

Mathieu Gruet

France

Oreste Gualillo

Spain

Andreas Guenther

Germany

Jesus Guinea

Spain

Erich Gulbins

Germany

Susan Guttentag

United States of America

Josef Guttmann

Germany

Tillie-Louise Hackett

Canada

Andrew Halayko

Canada

Keqi Han

China

Meilan Han

United States of America

Masayuki Hanaoka

Japan

Phil Hansbro

Australia

Jitka Stilund Hansen

Denmark

Corrine Hanson

United States of America

Christof Hauck

Germany

Gregory Hawkins

United States of America

Fengtian He

China

Irene Heijink

Netherlands

Matthias Held

Germany

Dirk Henrich

Germany

Darryl Hill

United Kingdom

Georgios Hillas

Greece

Ari Hirvonen

Finland

Jennifer Ho

Taiwan

Sandra Hoegl

Germany

Andrew Hoffman

United States of America

Olaf Holz

Germany

Robert Homer

United States of America

Amjad Horani

United States of America

Takahiko Horiguchi

Japan

Jay Horvat

Australia

Ildiko Horvath

Hungary

Guochang Hu

United States of America

Francois Huaux

Belgium

Alice Huertas

France

Wouter Huvenne

Belgium

Machteld Hylkema

Netherlands

Cristoforo Incorvaia

Italy

Satoru Ito

Japan

Kenji Izuhara

Japan

Daniel Jackson

United States of America

Jeffrey Jacobson

United States of America

Thomas Jagoe

Canada

Yong Ju Jang

South Korea

Luke Janssen

Canada

Andrew Paul Jarman

United Kingdom

Tuomas Jartti

Finland

Ilona Jaspers

United States of America

Zhilong Jiang

United States of America

Shengwei Jin

China

Zhi-Cheng Jing

China

Michelle John

United Kingdom

Jill Johnson

United Kingdom

Paul Jones

United Kingdom

Claude Jourdan-Le Saux

United States of America

Andreas Jung

Switzerland

Noor Kalsheker

United Kingdom

David Kamp

United States of America

Hannu Kankaanranta

Finland

Tobias Keck

Germany

Thomas Kennedy

United States of America

Huib Kerstjens

Netherlands

Farrah Kheramand

United States of America

Yoon-Keun Kim

South Korea

Greg King

Australia

Pascale Kippelen

United Kingdom

Loes Kistemaker

Netherlands

Yasuo Kizawa

Japan

Darryl Knight

Australia

Alan Knox

United Kingdom

Vladimir Koblizek

Czech Republic

Martin Kolb

Canada

Jay Kolls

United States of America

Masuo Kondoh

Japan

Mirjam Kool

Netherlands

Martina Korfei

Germany

Beata Kosmider

United States of America

Konstantinos Kostikas

Greece

Anastasia Kotanidou

Greece

Thomas Kovesi

Canada

Frank Kruyt

Netherlands

Vera Krymskaya

United States of America

Wolfgang Kuebler

Germany

Kuldeep Kumawat

Netherlands

Philipp Kümpers

Germany

Lisette Kunz

Netherlands

Ming-Ling Kuo

Taiwan

Katia Kvitko

Brazil

Jeff Kwon

United States of America

Vincent Lagente

France

Franco Laghi

United States of America

Lies Lahousse

Belgium

Irene Lang

Austria

Sven Larsson

Sweden

François Laurent

France

Geoff Laurent

Australia

William Lawson

United States of America

John Lay

United States of America

Aili Lazaar

United States of America

Chun Geun Lee

United States of America

Janet Lee

United States of America

Kang-Yun Lee

Taiwan

Hakon Leffler

Sweden

Rosa Lemmens-Gruber

Austria

Michael Levin

South Africa

Bing Li

China

Guiyuan Li

China

Triantafillos Liloglou

United Kingdom

Shizhang Ling

United States of America

Dominik Linz

Germany

Paulo Lisboa

United Kingdom

Fu-Tong Liu

United States of America

Gang Liu

China

Jie Liu

United States of America

Olle Löwhagen

Sweden

Marek Lommatzsch

Germany

Lucille London

United States of America

M Victorina Lopez Varela

Uruguay

Victorina Lopez Varela

Uruguay

Renaud Louis

Belgium

Stelios Loukides

Greece

James Loyd

United States of America

Zi-Qiang Luo

China

Harm Maarsingh

Netherlands

Roberto Machado

United States of America

Tania Maes

Belgium

Arnaud Mailleux

France

Nicholas Manzo

United States of America

Jian-Hua Mao

United States of America

Crystal Marconett

United States of America

Bernard Mari

France

Thomas Mariani

United States of America

Oreste Marrone

Italy

Shuichiro Maruoka

Japan

Jorge Maspero

Argentina

Kazuo Matsubara

Japan

Shin Matsuoka

Japan

Hiroto Matsuse

Japan

Gustavo Matute-Bello

United States of America

Thais Mauad

Brazil

Donald M. McDonald

United States of America

Michael McGoon

United States of America

Sharon McGrath-Morrow

United States of America

Pamela McShane

United States of America

Eckart Meese

Germany

Borna Mehrad

United States of America

Atul Mehta

United States of America

Davendra Mehta

United States of America

M. Asuncion Mejias

United States of America

Andrea Melani

Italy

Barbro Melgert

Netherlands

Massimo Miniati

Italy

Michael Minnicozzi

United States of America

Jordan Minov

Macedonia

Marc Miravitlles

Spain

Firdaus A A Mohamed Hoesein

Netherlands

Lyn Moir

Australia

Winfried Möller

Germany

Paolo Montuschi

Italy

Juan Moreno

Spain

Sarah Morgan

United States of America

Hideaki Moriyama

United States of America

Edward Morrisey

United States of America

Esmaeil Mortaz

Netherlands

Rory Morty

Germany

Robert M Mroz

Poland

**Joachim M**ü**ller-Quernheim**

Germany

Anil Mukherjee

United States of America

Veronika Müller

Hungary

John T Murchison

United Kingdom

Shigeo Muro

Japan

Lynne Murray

United Kingdom

Kari Nadeau

United States of America

Jay Nadel

United States of America

Rachel Nadif

France

Robert Naeije

Belgium

Yasutaka Nakano

Japan

Patrick Nana-Sinkam

United States of America

Nadia Nathan

France

Tim Nawrot

Belgium

Thao Nguyen

United States of America

Linda Nici

United States of America

Koichi Nishimura

United Kingdom

Yoshihiro Nishimura

Japan

Magnus Nord

Sweden

Benjamin Nwosu

United States of America

Benjamin Odry

United States of America

Toru Oga

Japan

Yoshiharu Ohno

Japan

Brian Oliver

Australia

Liam O'Mahony

Switzerland

Michael O'Reilly

United States of America

Larry Ostrowski

United States of America

Caroline Owen

United States of America

Alix Paget-Brown

United States of America

Robert Paine

United States of America

Lena Palmberg

Sweden

Kusum Pandit

United States of America

Angela Panoskaltsis-Mortari

United States of America

Gregorino Paone

Italy

Andriana Papaioannou

Greece

Jin-Ah Park

United States of America

Sean Parker

United Kingdom

Anand Patel

United States of America

Ian Pavord

United Kingdom

Girolamo Pelaia

Italy

Raymond Penn

United States of America

Tonio Pera

United States of America

Jesus Perez-Gil

Spain

Rogelio Perez-Padilla

Mexico

Leif Peterson

United States of America

Irina Petrache

United States of America

Alejandro Pezzulo

United States of America

Massimo Pifferi

Italy

Valéria Amorim Pires Di Lorenzo

Brazil

Francesco Pistelli

Italy

Fabio Pitta

Brazil

Venerino Poletti

Italy

Michael Polkey

United Kingdom

Jill Poole

United States of America

Nicolas Pottier

France

Sandeep Prabhu

United States of America

Y S Prakash

United States of America

Jeff Pretto

Australia

David Price

United Kingdom

Mirella Profita

Italy

Grzegorz Przybylski

Poland

Jennifer Quint

United Kingdom

Antoine Rabbat

France

Roberto Rabinovich

United Kingdom

Thirumalai Ramalingam

United States of America

Kathryn Ramsey

Australia

Sarah Rand

United Kingdom

Winfried Randerath

Germany

Sekhar Reddy

United States of America

John Reilly

United States of America

Niki Reynaert

Netherlands

Paul Reynolds

United States of America

Fabio Ricciardolo

Italy

Gerdt C Riise

Sweden

Roberto Rodriguez-Roisin

Spain

Paola Rogliani

Italy

Jesse Roman

United States of America

Debra Romberger

United States of America

Philippe Rosias

Netherlands

Mahmoud Rouabhia

Canada

Jeremie Roux

United States of America

Nikoletta Rovina

Greece

Lewis Rubin

United States of America

Clark Russell

United Kingdom

Erica Rutten

Netherlands

Sungwoo Ryoo

South Korea

Ruxana Sadikot

United States of America

Venkatesh Sampath

United States of America

Manuel Sanchez-de-la-Torre

Spain

Philip Sannes

United States of America

Shinji Sasada

Japan

Dedmer Schaafsma

Canada

Tom Schaberg

Germany

Bernhard Schlechta

Austria

Martina Schmidt

Netherlands

Bettina Schock

United Kingdom

Richard Schulz

Germany

Christian Schumann

Germany

Stefan Schumann

Germany

Chris Scotton

United Kingdom

Andrea Segreti

Italy

Petra Seidel

Switzerland

Sanjay Sethi

United States of America

Huahao Shen

China

Yuichiro Shindo

Japan

Janis Shute

United Kingdom

Nikos Siafakas

Greece

Salman Siddiqui

United Kingdom

Richard Sindelar

Sweden

Dave Singh

United Kingdom

Thomas Sisson

United States of America

Chrysanthi Skevaki

Greece

Kristopher Skwarski

United Kingdom

Patricio Smith

Chile

Hermelijn Smits

Netherlands

Sigrun Smola

Germany

Anita Spanjer

Netherlands

Mark Spears

United Kingdom

Eliot Spindel

United States of America

Martijn Spruit

Netherlands

Saranya Sridhar

United Kingdom

Bruce Stanton

United States of America

Karl Staples

United Kingdom

Katrina Steiling

United States of America

Michael Steiner

United Kingdom

Robert Stockley

United Kingdom

Clare Stokes

United Kingdom

Viranuj Sueblinvong

United States of America

Maria Sukkar

Australia

Yongchang Sun

China

Norbert Suttorp

Germany

Nicola Sverzellati

Italy

Erna Sziksz

Hungary

Clifford Taggart

United Kingdom

Osamu Taguchi

Japan

Masao Takata

United Kingdom

Tsutomu Tamada

Japan

Hui-Leng Tan

United Kingdom

Dale Tang

United States of America

Jun-Ming Tang

China

Suzana Erico Tanni

Brazil

Keith Tanswell

Canada

Laima Taraseviciene-Stewart

United States of America

Donald Tashkin

United States of America

Christian Taube

Netherlands

Klaus Tenbrock

Germany

Neil Thomson

United Kingdom

Ying Tian

United States of America

Renaud Tissier

France

Antoni Torres

Spain

Maria Tsoumakidou

Greece

Gerard M Turino

United States of America

Eleni Tzortzaki

Greece

Argyris Tzouvelekis

Greece

Paul Upton

United Kingdom

Joost G van den Aardweg

Netherlands

Maarten van den Berge

Netherlands

Rene van der Merwe

United Kingdom

Albert van der Vleit

United States of America

Arjan van Laarhoven

Netherlands

Pieter-Jan van Ooij

Netherlands

Jeroen Vanoirbeek

Belgium

Ruud Veldhuizen

Canada

Giovanni Viegi

Italy

Eszter Vladar

United States of America

Ross Vlahos

Australia

Claus Vogelmeier

Germany

Stephan von Gunten

Switzerland

Robert Voswinckel

Germany

Gerry Wagenaar

Netherlands

Benoit Wallaert

France

David Warburton

United States of America

Peter Wark

Australia

Jenora Waterman

United States of America

Henrik Watz

Germany

Guangwei Wei

China

Athol Wells

United Kingdom

Tobias Welte

Germany

Fu-Qiang Wen

China

Sally Wenzel

United States of America

Susan Wert

United States of America

Gunilla Westergren-Thorsson

Sweden

Guillaume Wettstein

France

Eric White

United States of America

Jim White

United States of America

Janine Wichmann

South Africa

Mark Wielpuetz

Germany

Peter Wijkstra

Netherlands

Tim Williamson

United States of America

David Wilson

United States of America

Aaron Winkler

United States of America

Brent Winston

Canada

Hubert Wirtz

Germany

Kenneth Witwer

United States of America

Jeremias Wohlschlaeger

Germany

Emiel F M Wouters

Netherlands

Ximei Wu

China

Danielle Wurzel

Australia

Masao Yamaguchi

Japan

Hideyuki Yamamoto

Japan

Ian Yang

Australia

Qi Yang

United States of America

Xin Yao

China

Kazuhiro Yasufuku

Canada

Junji Yodoi

Japan

Akihito Yokoyama

Japan

Robert P Young

New Zealand

Jason Yuan

United States of America

Elodie Zana-Taïeb

France

Paul Zarogoulidis

Greece

Hui Zeng

China

Huayan Zhang

United States of America

Ze Zhang

China

Jinshun Zhao

China

Weian Zhao

United States of America

Yutong Zhao

United States of America

Degui Zhi

United States of America

